# Effect of Elevated Temperature on Mechanical Properties and Shielding Performance of Magnetite–Serpentine Radiation-Proof Concrete

**DOI:** 10.3390/ma18122686

**Published:** 2025-06-06

**Authors:** Dan Wu, Zehua Liu, Zhenfu Chen, Qiongfang Wu, Qiuwang Tao

**Affiliations:** 1 School of Resources Environment and Safety Engineering, University of South China, Hengyang 421001, China; 2017000067@usc.edu.cn; 2 School of Civil Engineering, University of South China, Hengyang 421001, China; 2009000110@usc.edu.cn (Q.W.); taoqiuwang@usc.edu.cn (Q.T.); 3 China Nuclear Industry Key Laboratory of High-Performance Concrete, University of South China, Hengyang 421001, China; 4 Hunan Provincial Key Laboratory of High-Performance Special Concrete, University of South China, Hengyang 421001, China

**Keywords:** magnetite–serpentine concrete, high temperature, mechanical properties, shielding performance

## Abstract

High temperatures can induce a range of physical and chemical alterations in radiation-protective concrete, potentially compromising its strength and significantly diminishing its radiation shielding capabilities. Therefore, it is very important to study the high temperature performance of radiation-proof concrete to ensure its safety and stability in extreme environment. In this study, the magnetite–serpentine radiation-proof concrete is designed with magnetite as coarse aggregate, serpentine as fine aggregate, and Portland cement and granulated blast furnace slag as mixture. The apparent characteristics, mass loss, ultrasonic pulse velocity, mechanical properties, shielding performance, and correlation of this concrete were analyzed through experiments. The results show that the damage degree and relative wave velocity have a good correlation in evaluating the relative mass loss, linear attenuation coefficient, compressive strength, and tensile strength after high temperatures. The compressive strength at 800 °C is 12.2 MPa and the splitting tensile strength is 0.48 MPa; the linear attenuation coefficient of specimen at 800 °C is reduced to 80.9% of that at normal temperature. Meanwhile, penetrating cracks appeared at 600 °C and spalling phenomenon appeared at 800 °C, and better thermal stability and favorable mechanical properties and shielding performance also occurred; thus, suitable radioactive and high temperature environment was determined. The results could provide scientific guidance for nondestructive testing and performance evaluation of shielding structure materials.

## 1. Introduction

Radiation shielding concrete is frequently utilized in nuclear facilities, including nuclear power plants and repositories for nuclear waste. These facilities may face high-temperature environments during normal operation or in the event of an accident. For example, the pressure vessels and containment structures of nuclear power plants are often subjected to normal operating conditions at temperatures ranging from 60 to 120 °C. Particularly, radiation shielding concrete used in military engineering can experience surface temperatures rising to 900 °C or higher within 1 h when attacked by weapons [1]. High temperatures can initiate and propagate cracks within the concrete, consequently leading to a marked reduction in its strength, durability, and radiation shielding efficacy. Therefore, research on the high-temperature behavior of radiation shielding concrete to understand the effects and mechanisms of high temperatures is crucial for ensuring the safety and stability of radiation shielding concrete in practical applications and for safeguarding nuclear security.

Extensive experimental research has demonstrated that factors including concrete density, aggregate type, aggregate volume fraction [2,3], and particle size distribution within heavy concrete substantially affect the attenuation of gamma and neutron radiation [4]. Temperature directly affects the mechanical properties of concrete and high temperatures can markedly reduce its compressive strength and splitting tensile strength and may even lead to a complete loss of load-bearing capacity [5,6]. At the same time, the high temperature effects can reduce the radiation shielding ability of concrete such as serpentine [7], magnetite [8], barite [9], siderite [10], masoud, and others [11] indicate that the hydrogen content in serpentine plays a decisive role in neutron attenuation, while an increase in dolomite and asbestos content can reduce gamma radiation shielding performance. P. Tamayo and colleagues suggest that a 25% increase in density results in approximately a 10% increase in the linear attenuation coefficient of concrete [12]. Omran O.L et al. [13] and Mahmoud M.E et al. [14] believed that the particle size distribution and direction of particles had a great influence on the attenuation, and the probability of collision between the incident light and the heavy particles in the concrete plays a decisive role.

Studies have revealed that the incorporation of serpentine as coarse aggregate diminishes concrete workability and escalates water requirements, whereas the use of fine aggregates can ameliorate the microstructure and bolster the compressive strength of the concrete [15]. Magnetite concrete exhibits superior mechanical and shielding properties compared to barite, goethite, hematite [16], and limonite [17]. When barite and hematite replace serpentine at different ratios, the mechanical properties of the concrete are inferior to those of pure serpentine, with the 28-day compressive strength not reaching 30 MPa [18]. Pure cementitious materials exhibit the poorest resistance to gamma radiation [19]. Although the substitution of cement with fly ash improves the workability of hematite concrete, it reduces its compressive strength [20]. In contrast, the replacement of cement with granulated blast furnace slag not only improves the flowability and mechanical strength of concrete [21,22] but also reduces cracking behavior [23] and carbon emissions [24,25].

Magnetite is an iron oxide mineral that contains Fe^2+^ and Fe^3+^ ions in its structure and has a high atomic number. Serpentine has the chemical formula 3MgO•2SiO_2_•2H_2_O and contains hydroxyl groups in its structure and is rich in hydrogen atoms. It is evident that materials such as magnetite and serpentine are high-quality raw materials for radiation shielding concrete. Although there has been extensive research on shielding concrete made from single materials or combinations such as magnetite–barite [26] and barite–limonite [27], studies on radiation shielding concrete composed of magnetite–serpentine composite materials are scarce. In this paper, granulated blast furnace slag is used as admixture to replace part of Portland cement (P.O.42.5R), and the mixture ratio of strength grade 40 MPa magnetite–serpentine composite aggregate radiation-proof concrete is obtained by response surface regression analysis. Based on the tests of apparent characteristics, mass loss, ultrasonic pulse velocity, mechanical properties, and γ-ray shielding, the high-temperature mechanical properties and radiation shielding ability of composite aggregate radiation-proof concrete after different temperatures (24 °C, 100 °C, 200 °C, 300 °C, 450 °C, 600 °C, and 800 °C) are studied and analyzed. The results show that relative wave velocity have a linear correlation with the relative mass loss, linear attenuation coefficient, compressive strength, and tensile strength. The mechanical strength and linear attenuation coefficient of magnetite–serpentine composite concrete were 42.0 MPa and 0.236 cm^−1^ at room temperature (12.2 MPa and 0.189 cm^−1^ at 800 °C). Compared with the existing data related to serpentine, magnetite–serpentine has better mechanical strength and radiation shielding properties, which are suitable for concrete for shielding structures under the action of high temperature. The results of the study can provide scientific guidance for optimizing the design of concrete proportion and exploring the assessment and characterization of the degree of damage of concrete after high temperatures, as well as the design of shielding structures for buildings such as reactor vessels, nuclear waste placement, and treatment.

## 2. Materials and Methods

### 2.1. Materials

**Figure 1:**
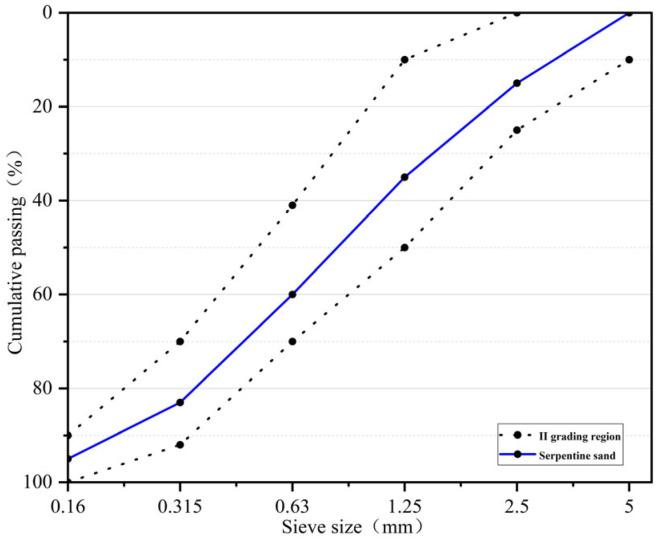
Screening curves of coarse aggregates.

**Figure 2:**
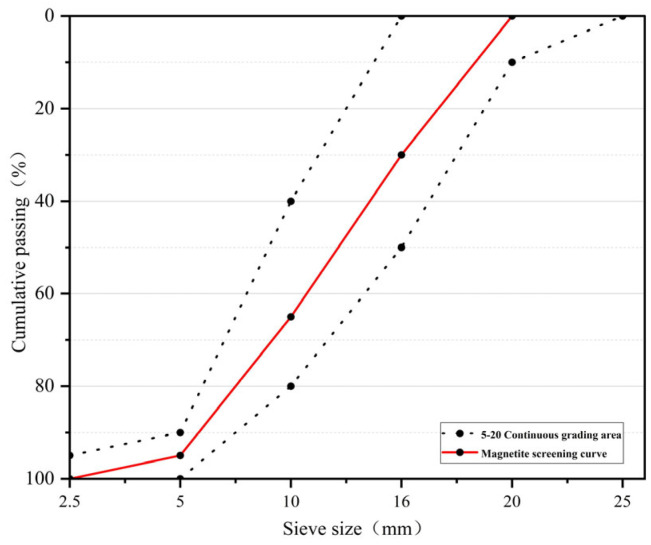
Screening curves of fine aggregates.

Portland cement (P.O.42.5R) in accordance with the Chinese standard GB175-2020 [28] was used for preparing concrete in this research. Granulated blast furnace slag (GBFS) was used as partial cement replacement material, with a density of 3100 kg/m^3^. The coarse aggregate used in this experiment was magnetite, with a particle size of 5–20 mm, the origin stone was washed and dried, crushed by a jaw crush, sifted by a standard square hole sieve in three specifications of 5–10 mm, 10–20 mm and above 20 mm, and the needles and flakes in the aggregate were removed. The fine aggregate was serpentinite, which was crushed from the origin stone into sand, with a particle size of 0.15–5 mm and a fineness modulus of 2.8, which was a well-graded medium sand. The aggregate screening curves are shown in Figure 1 and Figure 2. The chemical composition of cement, GBFS, magnetite, and serpentine are shown in Table 1, the basic properties of cement are shown in Table 2, and the basic properties of aggregates are shown in Table 3. Water reducer (CQJ-JSS02 polycarboxylate superplasticizer) and defoamer were provided by a company (produced by Gongyi Lanke Water Purification Material Co., Ltd., Gongyi, China), and the mixing water was ordinary tap water.

### 2.2. Mix Design

Single factor experiments were conducted to determine the optimal dosage of 30% by replacing cement with granulated blast furnace slag in proportions of 0%, 10%, 20%, 30%, and 40%, with the result shown in Table 4.

Taking the water-binder ratio, sand ratio, and water consumption as independent variables and 28 day compressive strength as the response value, a three-factor and three-level test scheme was designed by Box–Behnken in the Design-expert 13.0 software, as shown in Table 5. According to the GB/T 34008-2017 [29] and GB/T 50081-2019 [30] standard procedures, a total of 17 sets of experiments were conducted, and three parallel tests were performed in each set.

Based on the analysis of variance results, the model’s F value is 41.67, and the *p* value is less than 0.0001, indicating that the model is significant. Additionally, the R-squared (R2) value and adjusted R-squared (Adj-R2) value are 0.9817 and 0.9581, respectively, both close to 1. The difference between the Adj-R2 value and the Predicted R-squared (Pre-R2) value is 0.1805, which is less than 0.2, suggesting that the established model is reasonable. Through regression analysis, the fitting equation for the 28-day unconfined compressive strength is as follows:

Y = 566.85309 − 763.88571A − 687.0068B − 1.97835C − 671.42857AB + 1.5AC + 3.19048BC + 639.0A^2^ + 283.67347B^2^ + 0.000656C^2^ (1)

where Y is the compressive strength (MPa), A is the water-binder ratio, B is the sand ratio, and C is water consumption (kg/m^3^).

The RSM analysis determined the mix ratio of magnetite–serpentine composite aggregate radiation shielding concrete (hereinafter referred to as M_F_) as shown in Table 6. Based on the designed mix ratio in Table 6 and the basic provisions of GB/T 50082-2009 [31], 100 × 100 × 100 mm^3^ specimens were formed. The measured mass volume density of the specimens at room temperature is 2890 kg/m^3^, the slump is 107 mm, the compressive strength is 42.0 MPa, the ultrasonic velocity is 4.526 km/s, and the radiation shielding linear attenuation coefficient is approximately 0.236 cm^−1^. In accordance with GB/T 34008-2017, this design of radiation shielding concrete meets the R1 density grade, S3 slump grade, and the C40 design requirements. It also fulfills the requirements for neutron radiation on materials and gamma radiation shielding capability, while possessing good mechanical properties and structural compactness.

### 2.3. Test Method

The typical size of cubic specimen is 100 × 100 × 100 mm^3^ and prism is 100 × 100 × 300 mm^3^, which were prepared according to GB/T 50082-2009. The specimens were placed in a normal with a temperature of 20 ± 5 °C and a relative humidity > 50% for 24 h. Then the molds were removed and the specimens were marked. After that, the specimens were placed in a standard curing room with a temperature of 20 ± 2 °C and a relative humidity ≥ 95% for 28 days. During the test, the M_F_ sample was placed in an oven at 105 ± 5 °C for constant temperature drying for 24 h. After the oven was naturally cooled to normal temperatures, the sample was immediately put into the SX2-36-12-1300 °C box muffle furnace (produced by Xiangtan Xiangyi Instrucment Co. Ltd., Xiangtan, China), heated to the target temperature (100 °C, 200 °C, 300 °C, 450 °C, 600 °C, and 800 °C) at a rate of 3.5 °C/min, and the target temperature was kept constant for 1 h, with natural cooling to a normal temperature in the furnace.

#### 2.3.1. Apparent Characteristic Test

Observe and record the surface color and macroscopic texture of the 100 × 100 × 100 mm^3^ specimens after demolding. After high-temperature testing and cooling to room temperature, remove the samples to observe any changes in color and cracking conditions. This process is repeated for all six target temperatures.

#### 2.3.2. Mass Loss Test

The 100 × 100 × 100 mm^3^ M_F_ sample was weighed and placed in an oven at 105 ± 5 °C for constant temperature drying for 24 h. After the oven was naturally cooled to normal temperatures, the sample was taken out and weighed. After the high-temperature test cools to room temperature, remove the sample from the cooling furnace and weigh it. Seal the specimen with cling film for future testing. This process is repeated for all six target temperatures.

#### 2.3.3. Ultrasonic Nondestructive Testing

**Figure 3:**
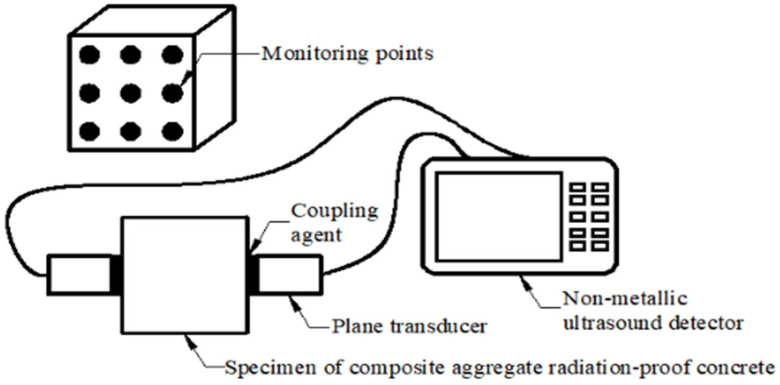
Schematic diagram of ultrasonic nondestructive testing.

The specimen size was 100 × 100 × 100 mm^3^ cube. The ZBL-U520(510) non-metallic ultrasonic detector (produced by Beijing Zbl Science Technology Co., Ltd., Beijing, China) was used in the test. Referring to the CSCS21-2000 [32], ultrasonic nondestructive testing was carried out on specimens with different temperatures and thicknesses before and after high temperatures, as shown in Figure 3 below.

#### 2.3.4. Mechanical Property Test

**Figure 4:**
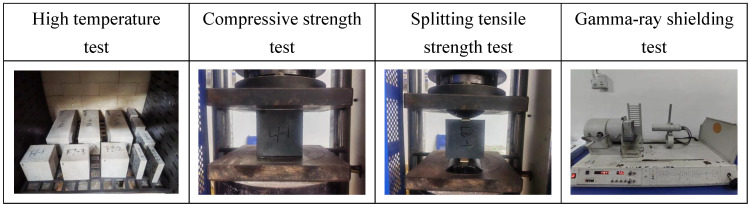
Test schematic representation.

Using WAW-EY1000C microcomputer (produced by Jinan Testing Machine Factory, Jinan, China) to control the electro-hydraulic servo universal testing machine, referring to the GB/T50081-2019, the compressive tests and static splitting tensile tests were carried out on 100 × 100 × 100 mm^3^ cubic specimens, and the axial compressive strength tests were carried out on 100 × 100 × 300 mm^3^ specimens, as shown in Figure 4 below.

#### 2.3.5. γ-Ray Shielding Performance Test

**Figure 5:**
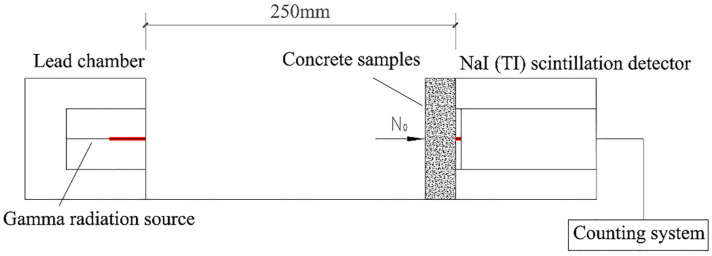
Gamma-ray shielding test diagram.

M_F_ specimens for the γ-ray shielding test were prepared as blocks with a section size of 100 × 100 mm^2^ and different thicknesses (22 mm, 47 mm, 69 mm, 100 mm, 122 mm). The 22 mm and 47 mm specimens were cut from 100 mm cubic specimens, while the 69 mm and 122 mm specimens were obtained by stacking them. The shielding performance of M_F_ was carried out on a BH1326 nuclear physics test platform, in which the γ-ray radiation source used ^137^Cs radioisotope with photon energy of 0.662 Mev. Each specimen was evenly divided into 9 different test points, and each test point was exposed to γ-ray radiation for 60 s. After the test, the instrument automatically counted. The γ-ray shielding performance was evaluated by the linear attenuation coefficient, half value layer, and ten value layer. The schematic diagram of the γ-ray shielding test is shown in Figure 4 and Figure 5.

## 3. Results

### 3.1. Apparent Characteristic Analysis

**Figure 6:**
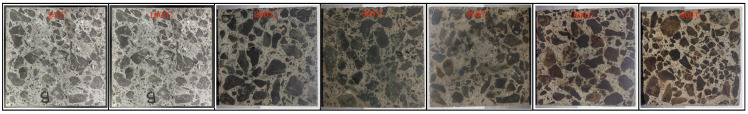
Appearance characteristics photos after different temperatures.

Figure 6 shows the apparent characteristic changes in the M_F_ specimens before and after high temperatures. As shown in the figure, at 100 °C–300 °C, the cement paste is dark gray, the aggregate remains unchanged, and there are no cracks in the sample; at 450 °C, the cement paste is dark gray, some aggregate turns brown, cracks begin to appear but are few and not wide, mainly distributed in fine aggregate and cementitious materials; at 600 °C–800 °C, the cement paste turns gray white, the aggregate turns brown, wide cracks and through cracks appear, and they mainly form at the interface between cementitious materials and aggregate. At 800 °C, a small amount of spalling occurs.

### 3.2. Mass Loss

**Figure 7:**
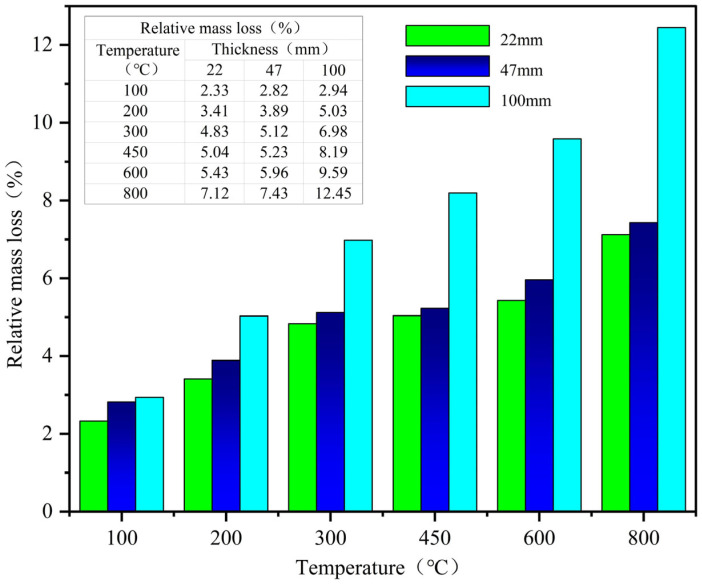
Relative mass loss of concretes at elevated temperatures.

According to Equation (2), the mass loss rate of M_F_ before and after different temperatures was calculated, and the average value of three specimens was taken as the test result, as shown in Figure 7 below.

(2) $$m_{s}=\frac{m_{0}-m_{T}}{m_{0}}\times 100\%$$

where

*m_s_* is the mass loss rate.

*m*_0_ is the mass of the specimen before different temperatures.

*m_T_* is the mass of the specimen after different temperatures.

As shown in Figure 7, the mass loss rate of M_F_ increases as the temperature rises. At 100 °C–300 °C, the mass loss rate increases linearly. At 300 °C–600 °C, the mass loss rate slows down, and the change range of 22 mm and 47 mm specimens is within 1%. The mass loss rate of 100 mm specimen reaches 12.45% at 800 °C. According to the data graph, it can be seen that the mass loss rate of specimen at 600 °C–800 °C is most affected by temperature, followed by 100 °C–300 °C, and at 300 °C–600 °C, it has a relatively small influence.

### 3.3. Ultrasonic Nondestructive Test

**Figure 8:**
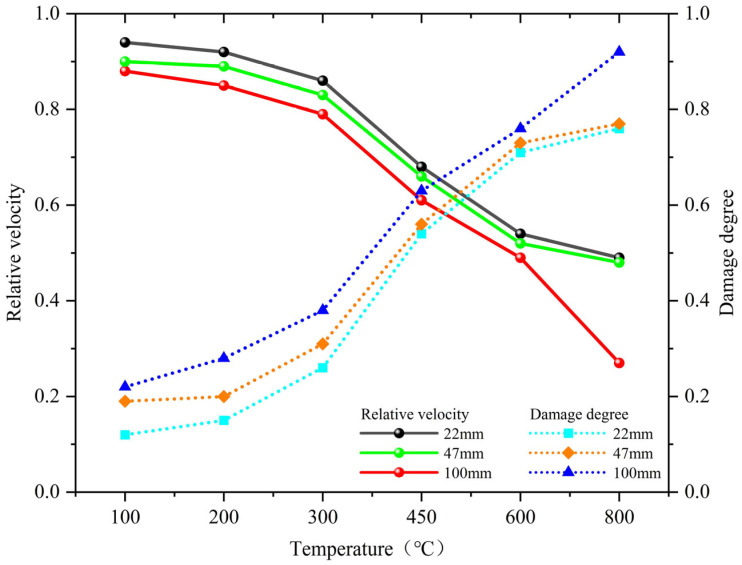
Relationship between varying thicknesses relative to velocity, damage degree, and temperature.

In this experiment, the relative wave velocity and damage degree were used as evaluation parameters, as shown in Equations (3) and (4). The results of relative wave velocity and damage degree of M_F_ at different temperatures are shown in Figure 8.

V_R_ = V_T_/V_0_ (3)

D = 1 – (V_T_/V_0_)^2^ (4)

where

V_R_ is the relative wave velocity.

V_T_ is the wave velocity of the specimen after different temperatures, km/s.

V_0_ is the wave velocity of the specimen before different temperatures, km/s.

D is the damage degree of M_F_.

Figure 8 shows that the relative wave velocity and damage degree of M_F_ are significantly affected by temperature. With the increase in temperature, the relative wave velocity of specimens with different thicknesses decreases, and the damage degree increases. At 100 °C–300 °C, the relative wave velocity and damage degree change relatively slowly. At 300 °C–600 °C, the decline slope of relative wave velocity and the increase slope of damage degree both increase. The relative wave velocity of the 100 mm specimens at 800 °C was 0.27 and the damage degree was 0.92. Furthermore, as shown in Figure 8, with the increase in distance measurement, the relative wave velocity of M_F_ decreases and the damage degree increases.

### 3.4. Mechanical Properties

#### 3.4.1. Mechanical Properties at Normal Temperatures

**Figure 9:**
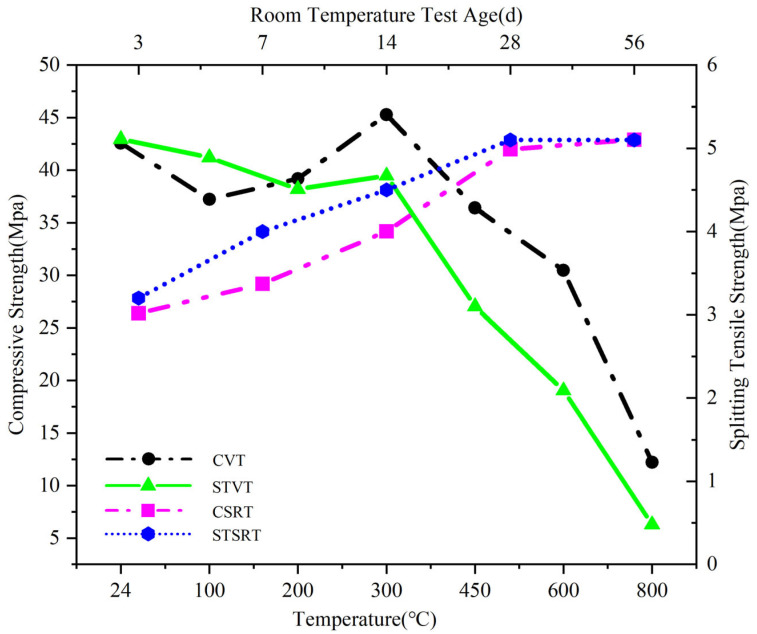
Compressive strength and splitting tensile strength at room and different temperatures. (CVT: compressive strength at varying temperatures; STVT: splitting tensile strength at varying temperatures; CSRT: compressive strength of room temperature at different curing ages; STSRT: splitting tensile strength of room temperature at different curing ages).

From the CSRT and STSRT curves in Figure 9, it can be seen that the compressive strength and splitting tensile strength of M_F_ increase with age under the standard curing conditions. At the age of 3 days, the compressive strength and splitting tensile strength both increase rapidly, but gradually slow down in the later stage. At 28 days, the strength is basically stable, with a compressive strength of 42.0 MPa and a tensile strength of 5.1 MPa. At 56 days, the compressive strength increases by 2.1% on the basis of 28 days, while the tensile strength remains unchanged at 5.1 MPa. It shows that the hydration rate under standard curing conditions is fast in the first 3 days after M_F_ casting, and the hydration reaction under standard curing conditions of 28 days is basically over. It is similar to the hydration law of ordinary crushed stone Portland cement.

#### 3.4.2. Compressive Strength Under Different Temperatures

From the CVT curve in Figure 9, it can be seen that the compressive strength shows a decreasing trend at 24 °C–100 °C and an increasing trend at 100 °C–300 °C, and the compressive strength at 300 °C is greater than that at normal temperatures. After 300 °C, the compressive strength continues to decrease, and after 600 °C, the rate of decrease is the fastest.

#### 3.4.3. Splitting Tensile Strength Under Different Temperatures

According to the STVT curve, the splitting tensile strength decreased nearly linearly at 24 °C–200 °C, and increased slightly at 200 °C–300 °C. The strength at 300 °C decreased by 8.6% compared with the initial strength and increased by 3.1% compared with the strength at 200 °C. The strength decreased continuously after 300 °C, and decreased the most at 600 °C–800 °C.

### 3.5. γ-Ray Shielding Performance

**Figure 10:**
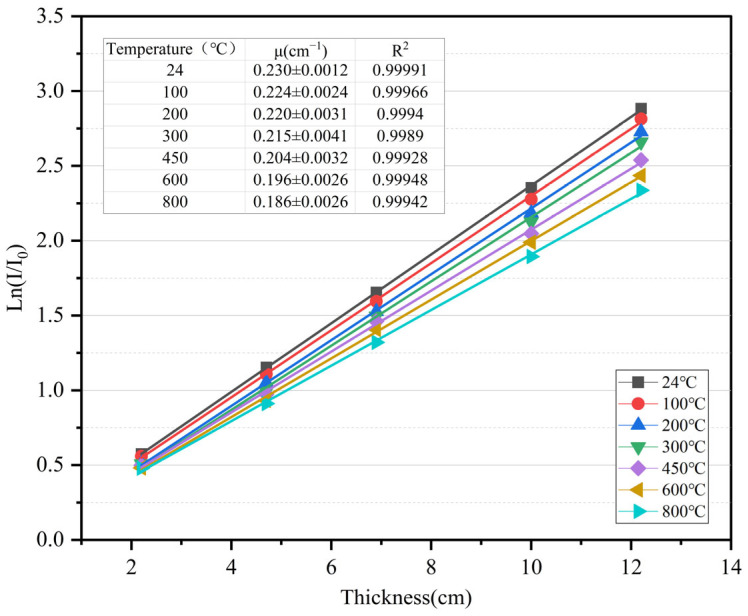
The relationship between M_F_ thickness and ln(I/I_0_).

**Figure 11:**
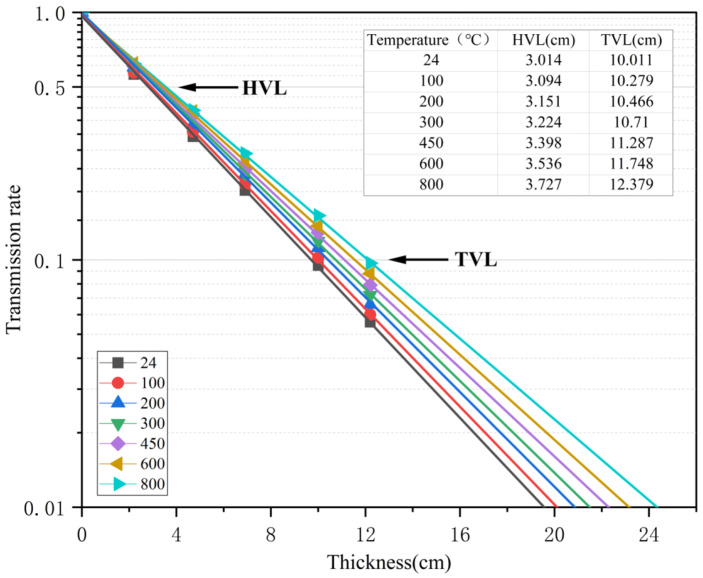
Relationship between gamma-ray transmission rate and concrete thickness.

According to Equations (5) and (8), the relationship among ln(*I*/*I*_0_), γ-ray transmittance, and specimen thickness was linearly fitted by least square method. According to Beer–Lambert law, the slope of the fitting line between ln(*I*/*I*_0_) and the *x* axis was a linear attenuation coefficient. The results are shown in Figure 10 and Figure 11.

(5) $$\mu =\frac{1}{x}\ln\frac{I_{0}}{I}$$

(6) $$HVL=\frac{\ln2}{\mu }=\frac{0.693}{\mu }$$

(7) $$TVL=\frac{\ln10}{\mu }=\frac{2.303}{\mu }$$

(8) $$\begin{array}{cc} Transmission & rate=\frac{I}{I_{0}}=e^{-\mu x} \end{array}$$

where

*μ* is the linear attenuation coefficient (unit: cm^−1^) of radiation shielding concrete under γ-ray irradiation with photon energy of 0.662 Mev.

*I* is the exposure rate (also known as the exposure count rate) of γ-rays after passing through the radiation shielding concrete specimen with a thickness of *x.*

*I*_0_ is the exposure rate when there is no radiation shielding concrete specimen.

*x* is the thickness of the concrete specimen (unit: cm).

*HVL* is the thickness of the specimen when the radiation intensity decays to 50% of the initial intensity.

*TVL* is the thickness of the specimen when the radiation intensity decays to 10% of the initial intensity.

Transmission rate is the γ-ray transmittance.

Figure 10 shows that the anti-radiation ability of M_F_ is positively correlated to the thickness of the specimen under the same temperature. The thicker the specimen, the stronger the anti-radiation ability. The γ-ray shielding ability of 12.2 cm specimen at normal temperatures is about five times that of the 2.2 cm specimen, and with the increase in thickness, the anti-radiation ability of the material is more affected by the temperature. Under the same thickness, the linear attenuation coefficient is inversely related to the test temperature. The higher the temperature, the lower the linear attenuation coefficient. The linear attenuation coefficient of the specimen at 800 °C is reduced to 80.1% of that at normal temperatures.

From Figure 11, it can be seen that when the temperature is constant, the γ-ray transmittance is inversely related to the thickness of the specimen, and the thicker the specimen, the lower the transmittance. When the thickness of the specimen is constant, the γ-ray transmittance is positively correlated with the temperature, and the higher the temperature, the greater the transmittance. From the calculation of Equations (6) and (7), it can be known that the thicknesses of the half-layer and the ten-layer values at normal temperatures are 3.014 cm and 10.011 cm, respectively. The thicknesses of the half-layer and the ten-layer values at 100 °C, 200 °C, 300 °C, 450 °C, 600 °C, and 800 °C are 102.7%, 104.5%, 107.0%, 112.7%, 117.3%, and 123.7% of that at 24 °C, respectively.

## 4. Discussion

### 4.1. Apparent Characteristic Analysis

The reasons for the changes in apparent characteristics shown in Figure 6 are due to several factors. On one hand, it is a result of water loss and dehydration of the cement paste, as well as the oxidation of magnetite aggregates and the decomposition of CaCO_3_ at high temperatures [33,34]. According to XRD tests, the oxidation of elements such as iron and magnesium mainly occurs at temperatures of 600 °C and above. On the other hand, thermal expansion and contraction caused by heat effects lead to internal stresses, which result in cracking after exposure to high temperatures [35]. Lastly, the large volume occupied by aggregates in concrete means that thermal conductivity is primarily influenced by the mineral composition and porosity. Under high temperature conditions, the non-uniform deformation between aggregates and mortar leads to the formation of cracks [36].

### 4.2. Mass Loss Analysis

**Figure 12:**
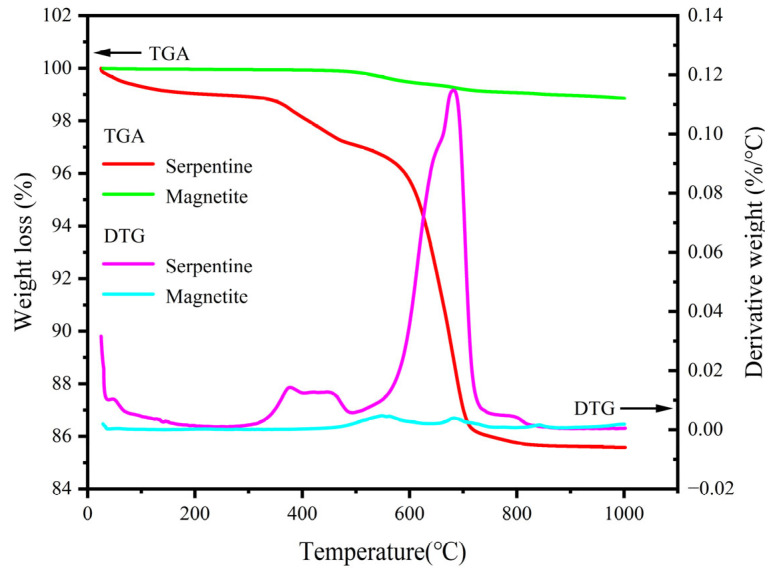
TGA and DTG curves for magnetite and serpentine aggregate.

After analysis, it is believed that when the temperature is around 100 °C, M_F_ is mainly the evaporation of free water or adsorbed water. Examined from the mineralogical point of view, serpentine, as a magnesium-bearing silicate mineral with a layered or fibrous crystal structure, is capable of containing a high amount of water of crystallization [11], and this water of crystallization is gradually lost at 100 °C–300 °C, resulting in mass loss. The rapid mass loss at 100 °C–300 °C is mainly due to the decomposition of calcium silicate hydrate (C-S-H) bound water and the evaporation of gel water. The mass loss rate slows down at 300 °C–600 °C, mainly due to the loss of chemically bound water, such as crystal water loss and dehydration and decomposition of calcium hydroxide [37,38,39]. However, although serpentine aggregates are rich in bound water, they have good thermal stability and only show significant mass loss above 600 °C [16,40]; magnetite maintains phase stability up to the temperature of 570 °C [9]. The TGA/DTG curves of magnetite and serpentine from Figure 12 confirm this phenomenon. At 600 °C–800 °C, the main cause of concrete mass loss is the escape of CO_2_ gas formed by the decomposition of calcium carbonate [41] and the decomposition of serpentine bound water.

### 4.3. Ultrasonic Detection Analysis

The results are mainly due to the fact that high temperature directly leads to an increase in internal pores and crack width in M_F_. When ultrasonic waves propagate to the cracks or holes generated by high temperature, phenomena such as reflection, detour, attenuation, and superposition occur, resulting in a decrease in propagation speed. After analysis, it is believed that at 100 °C–300 °C, the internal structure of the specimen becomes denser and new cracks are less developed, mainly due to the secondary hydration of M_F_ under high temperatures, which alleviates high-temperature damage to some extent. Cracks begin to appear at 300 °C–600 °C, and as the temperature increases, the cracks become larger and more. The damage caused by the high temperature effect plays a decisive role in reducing the ultrasonic velocity. At 800 °C, penetrating cracks appear, and the specimen deteriorates severely and becomes loose, resulting in a sharp decrease in ultrasonic velocity.

The experimental phenomena are similar to those of polypropylene fiber concrete [42], slag fly ash composite ordinary concrete [43], magnetite and barite radiation shielding concrete [44], and steel fiber barite concrete [45] under different high temperatures. According to the ultrasonic propagation characteristics, on the one hand, as the distance measurement increases, the sound absorption becomes stronger, leading to the gradual weakening of ultrasonic intensity and the slow consumption of energy. On the other hand, due to the increase in distance measurement, the factors affecting ultrasonic propagation increase and the probability of sound wave attenuation increases.

### 4.4. Analysis of Mechanical Properties 

#### 4.4.1. Effect of Thermal Gradients on Compressive Strength

The analysis of the experimental phenomena showed that the evaporation of the adsorbed water at 24 °C–100 °C mainly caused the increase in the internal porosity of the concrete, resulting in the decrease in the compressive strength. At 100 °C–300 °C, the increase in compressive strength was due to the late volcanic ash effect of granulated blast furnace slag [22] and the gap formed in the early stage, which provided a channel for steam overflow inside the specimen [46]. Under standard curing conditions, the hydration rate of granulated blast furnace slag powder was slower than that of cement clinker. After 28 days, there was dehydrated granulated blast furnace slag powder in the M_F_ [47].

As the temperature increased, water vapor promoted the hydration reaction of dehydrated granulated blast furnace slag to continue under steam pressure conditions. In addition, granulated blast furnace slag can reduce voidage and void volume [48], enhance the compactness of the interface transition zone, refine the concrete void structure [22,49], and enhance the internal friction of the concrete material [47], thereby improving the compressive strength. After 300 °C, the influence of thermal expansion on aggregates and mortar continued to increase. Due to the non-synergistic deformation of aggregates and mortar, microcracks were formed at the interface between the two phases [50] and the internal damage of concrete was more severe. In particular, after 450 °C, the hydrated calcium silicate, calcium aluminate, calcium hydroxide, which played a skeleton role in the cement stone, were thermally decomposed, leading to endothermic reactions and a rapid increase in void content [51]. In addition, thermal stress also causes a continuous decrease in strength.

#### 4.4.2. Effect of Thermal Gradients on Splitting Tensile Strength

The evolution of splitting tensile strength was that before 200 °C, both cement stone and mortar were in a state of thermal expansion, and the expansion of material components dominated [1], which conformed to the external tensile force and reduced the tensile strength. At 200 °C–300 °C, the cement stone began to shrink, promoting a more compact internal structure. Moreover, the temperature strengthening effect was higher than the temperature softening effect at 200 °C–300 °C [52], resulting in a rebound in tensile strength. After 300 °C, the thermal damage caused by microcracks inside the concrete became more obvious. The higher the temperature, the greater the pore pressure of the dense structure inside the M_F_, the more significant the impact on tensile strength, and the greater the loss of tensile strength. At the same time, the physical and chemical changes caused by high temperature in M_F_ significantly reduced the fracture energy of materials, leading to a rapid decrease in the tensile strength of M_F_ [53].

Compared to the compressive strength, it increased at 200 °C, mainly because the secondary hydration products under high temperature can fill some of the void structure and enhance the internal skeleton effect of the concrete but fail to form a good bonding effect with the materials around the voids, thus failing to generate energy to resist fracture. Overall, the loss rate of splitting tensile strength was relatively slow at 24 °C–300 °C and the decreases were not more than 10%. After 300 °C, there were significant changes. Compared with the strength at normal temperatures, the strength at 450 °C, 600 °C, and 800 °C decreased by 39.3%, 57.3%, and 89.4%, respectively.

### 4.5. γ-Ray Shielding Performance

γ-ray is composed of photons, which belongs to electromagnetic radiation, and its velocity is the same as that of light. The radiation shielding of concrete is affected by complex factors, depending on the material type, density, thickness [26], radiation strength [54], etc. When photons pass through matter, there are mainly photoelectric effect, Compton effect, and electron pair generation effect. It can be seen from the analysis of the test data that when the γ-ray energy is constant, the γ-ray collides with the inner electron or the outer electron of the atom, and the photon energy is transmitted or absorbed to reduce the energy of the secondary emission. At the same time, the presence of heavy elements such as iron increases the chance of secondary absorption of γ-ray. Therefore, the thicker the specimen, the greater the frequency of the Compton effect and photoelectric effect, the lower the γ-ray transmittance, and the better the shielding performance of the specimen [10,55].

With the increase in temperature, the increase in water loss leads to the decrease in specimen density, resulting in a decrease in the photon absorption ability, thereby weakening the photoelectric effect and reducing the radiation shielding ability [11,15,56]. At the same time, the increases in voidage and crack width caused by high temperatures lead to the decrease in radiation protection performance [7,57], and the thicker the specimen is required to be to shield the same radiation intensity, which brings adverse effects on the overall skeleton structure and working performance of the material.

### 4.6. Correlation Analysis

**Figure 13:**
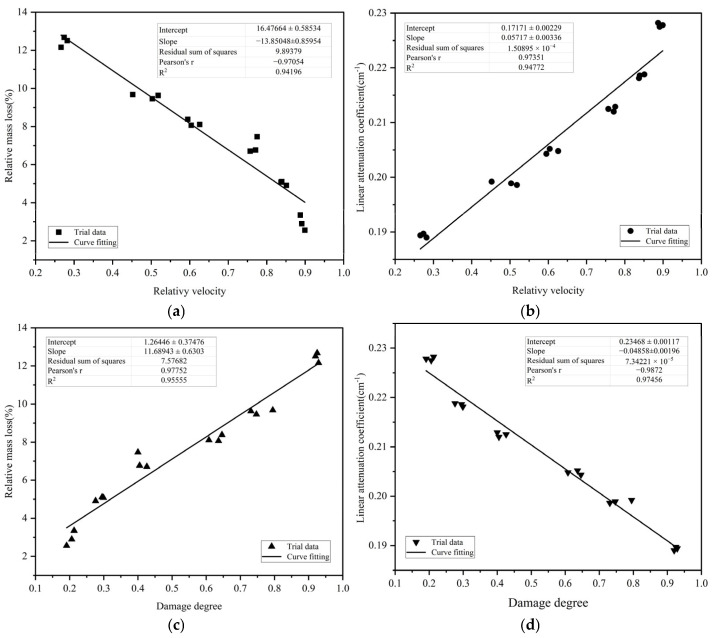
(**a**) Relationship between relative mass loss and relative velocity; (**b**) relationship between linear attenuation coefficient and relative velocity; (**c**) relationship between relative mass loss and damage degree; (**d**) relationship between linear attenuation coefficient and damage degree.

Taking a 100 mm specimen as an example, Figure 13 shows the experimental data and theoretical curves of relative mass loss and linear attenuation coefficient of the M_F_ specimen with the relative wave velocity and damage degree, respectively. The regression analysis is conducted by using a first-order polynomial fitting function, and the obtained fitting results are shown in Equations (9)–(12). According to the analysis of the fitting results, it is considered that the effect of damage degree is relatively good to evaluate the relative mass loss and linear attenuation coefficient after different temperatures.

(1) Relationship between relative mass loss and relative wave velocity, as shown in Figure 13a.

y = −13.85048x + 16.47664  R^2^ = 0.94196 (9)

(2) Relationship between linear attenuation coefficient and relative wave velocity, as shown in Figure 13b.

y = 0.05717x + 0.17171  R^2^ = 0.94772 (10)

(3) Relationship between relative mass loss and damage degree, as shown in Figure 13c.

y = 11.68943x + 1.26446  R^2^ = 0.95555 (11)

(4) Relationship between linear attenuation coefficient and damage degree, as shown in Figure 13d.

y = −0.04858x + 0.23468  R^2^ = 0.97456 (12)

**Figure 14:**
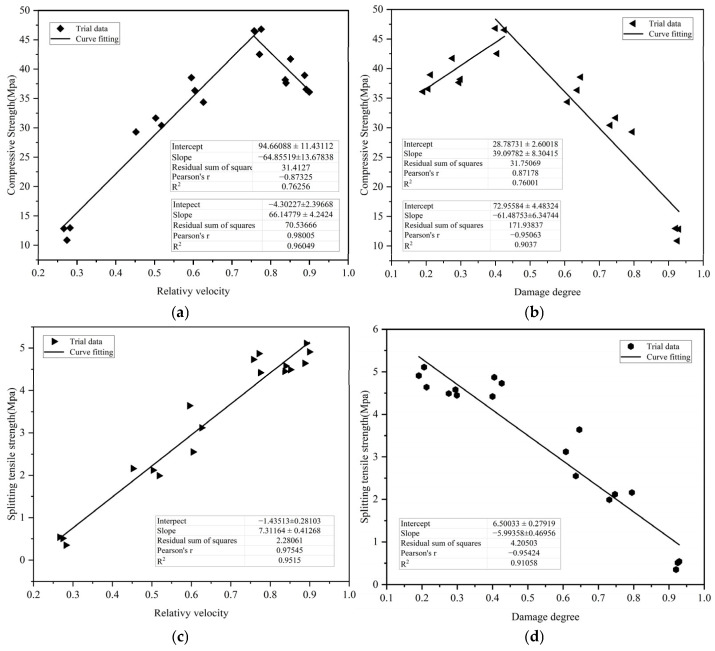
(**a**) Relationship between compressive strength and relative velocity; (**b**) relationship between compressive strength and damage degree; (**c**) relationship between splitting tensile strength and relative velocity; (**d**) relationship between splitting tensile strength and damage degree.

Figure 14 shows the experimental data and theoretical curves of compressive strength and splitting tensile strength of the M_F_ specimen with the relative wave velocity and damage degree, respectively. The regression analysis is also conducted by using a first-order polynomial fitting function, and the obtained fitting results are shown in Equations (13)–(16). It can be seen from Figure 14 that the overall effect of splitting tensile strength fitting is relatively good. Comparing the fitting results of relative wave velocity and damage degree, it is relatively optimal to evaluate the compressive strength and splitting tensile strength after different temperatures by using relative wave velocity.

(1) Relationship between compressive strength and relative wave velocity after different temperatures, as shown in Figure 14a.

(13) $$y=\left\{\begin{array}{c} -64.85519x+94.66088\;\;\;\;(100\;{}^{\circ}C\;\leq \;T\;\leq \;300\;{}^{\circ}C)\;\;\;\;R^{2}=0.76256\\ 66.14779x-4.30227\;\;\;\;\left(300\;{}^{\circ}C\leq T\leq 800\;{}^{\circ}C\right)\;\;\;\;R^{2}=0.96049\;\;\;\;\; \end{array}\right.$$

(2) Relationship between compressive strength and damage degree after different temperatures, as shown in Figure 14b.

(14) $$y=\left\{\begin{array}{c} 39.09782x+28.78731\;\;\;\;(100\;{}^{\circ}C\;\leq \;T\;\leq \;300\;{}^{\circ}C)\;\;\;\;R^{2}=0.76001\\ -61.48753x+72.95584\;\;\;\;(300\;{}^{\circ}C\;\leq \;T\;\leq \;800\;{}^{\circ}C)\;\;\;\;R^{2}=0.9037 \end{array}\right.$$

(3) Relationship between splitting tensile strength and relative wave velocity after different temperatures, as shown in Figure 14c.

y = 7.31164x − 1.43513  R^2^ = 0.9515 (15)

(4) Relationship between splitting tensile strength and damage degree after different temperatures, as shown in Figure 14d.

y = −5.99358x + 6.50033  R^2^ = 0.91058 (16)

## 5. Conclusions

This study innovatively adopts the refined design research method of response surface method to optimize the mix ratio with multiple factors, which improves the efficiency of mix ratio design. The mass loss, ultrasonic velocity, and mechanical and shielding properties of magnetite–serpentine composite aggregate system radioprotective concrete are investigated, which breaks through the limitations of the traditional research on a single aggregate (e.g., barite, magnetite), characterizes the shielding properties with multiple parameters, and establishes a mathematical model of high-temperature damage evaluation indexes, which fills in the gap in the research on the composite aggregate radioprotective concrete in high-temperature scenarios, and emphasizes the importance of considering temperature conditions when designing and evaluating the shielding structures, emphasizing the importance of considering temperature conditions when designing and evaluating shielding structures. The specific conclusions are as follows:

(1) The mass loss rate of magnetite–serpentine radiation shielding concrete increased with the increase in temperature. In the test temperature range, the mass loss rate of specimens at 600 °C−800 °C was most affected by temperature, as well as penetrating cracks. The mass loss rate of 100 mm specimen reached 12.45% at 800 °C and caused spalling phenomenon.
(2) The compressive strength of magnetite–serpentine radiation-proof concrete was increased by 20.3% when mixed with 30% of granulated blast furnace slag to replace cement after 600 °C, the mechanical property rate of decrease is the fastest, the compressive strength at 800 °C was 12.22 MPa, and the splitting tensile strength was 0.48 MPa.
(3) The relative wave velocity of magnetite–serpentine radiation shielding concrete is inversely correlated with temperature and distance measurement, while the damage degree is positively correlated. The relative wave velocity of the 100 mm specimen at 800 °C was 0.27 and the damage degree was 0.92.
(4) The thicker the specimen, the stronger the radiation protection ability of magnetite–serpentine radiation shielding concrete. The higher the temperature, the lower the linear attenuation coefficient. The γ-ray shielding ability of 12.2 cm specimen was about 5 times that of 2.2 cm specimen at normal temperatures. At 800 °C, the linear attenuation coefficient of the specimen decreased to 80.1% of that at normal temperatures. The half-layer and ten-layer values at 100 °C, 200 °C, 300 °C, 450 °C, 600 °C, and 800 °C were 102.7%, 104.5%, 107.0%, 112.7%, 117.3%, and 123.7% of those at normal temperatures, respectively.
(5) The damage degree and relative wave velocity have a linear correlation with the relative mass loss, linear attenuation coefficient, compressive strength, and tensile strength, which provide a foundation for the quantitative detection of concrete thermal damage by ultrasonic properties.

## Figures and Tables

**Table 1:** Chemical compositions of cement, GBFS, magnetite, and serpentine (wt.%).

Oxides	Chemical Composition(wt%)			
	Cement	GBFS	Magnetite	Serpentine
Na_2_O	0.24	-	0.41	-
MgO	1.77	6.01	9.62	51.5
Al_2_O_3_	7.53	17.7	12.99	1.26
SiO_2_	23.73	34.5	17.19	37.42
P_2_O_5_	-	-	0.41	0.04
SO_3_	3.87	1.64	0.83	0.06
K_2_O	1.31	-	0.42	0.14
CaO	56.45	34.0	2.86	0.65
TiO_2_	-	-	9.96	-
Cr_2_O_3_	-	-	0.84	0.31
Fe_2_O_3_	4.16	1.03	43.78	8.01
LOI	0.94	5.12	0.69	0.61

**Table 2:** Basic properties of cement.

Specific Surface Area (m^2^/kg)	Setting Time (min)		Compressive Strength (MPa)		Flexural Strength (MPa)	
	Initial Setting	Final Setting	3d	28d	3d	28d
333	212	268	28.4	52.0	5.4	8.4

**Table 3:** Basic properties of magnetite and serpentine.

Material Type	Apparent Density (kg/m^3^)	Crush Value index (%)	Absorption (%)	Moisture Content (%)
Magnetite	4400	0.35	0.2	0.1
Serpentine	2520	-	7.1	0.7

**Table 4:** Compressive strength of concrete with different proportions granular blast furnace slag.

Concrete Code	GBFS (%)	7d (MPa)	28d (MPa)
MS_0_	0	24.4	34.9
MS_1_	10	26.5	37.1
MS_2_	20	27.0	38.9
MS_3_	30	29.2	42.0
MS_4_	40	26.8	37.6

**Table 5:** Experimental factors and levels of coding.

Impact Factors	Code	Standardized Value			Starting Value		
		Low	Medium	High	Low	Medium	High
Water-binder ratio	A	−1	0	1	0.4	0.45	0.5
Sand ratio	B	−1	0	1	0.29	0.325	0.36
Water consumption	C	−1	0	1	220	235	250

**Table 6:** Mix proportions of concrete.

Concrete Type	W/B	Mix Proportion (kg/m^3^)						
		Cement	Magnetite	Serpentinite	GBFS	Water	Water-Reducing Agent	Defoaming Agent
M_F_	0.4	403	1409	641	173	230	1.7	8.6

## Data Availability

The original contributions presented in this study are included in the article. Further inquiries can be directed to the corresponding authors.
